# Diagnosis of Lung Cancer Using Endobronchial Ultrasonography Image Based on Multi-Scale Image and Multi-Feature Fusion Framework

**DOI:** 10.3390/tomography11030024

**Published:** 2025-02-27

**Authors:** Huitao Wang, Takahiro Nakajima, Kohei Shikano, Yukihiro Nomura, Toshiya Nakaguchi

**Affiliations:** 1 Department of Medical Engineering, Graduate School of Science and Engineering, Chiba University, Chiba 263-8522, Japan; wanghuitao@chiba-u.jp; 2 Department of General Thoracic Surgery, Dokkyo Medical University, Mibu 321-0293, Japan; t-nakajima@dokkyomed.ac.jp; 3 Department of Respirology, Graduate School of Medicine, Chiba University, Chiba 260-8670, Japan; shika.v.a.r.1107@gmail.com; 4 Center for Frontier Medical Engineering, Chiba University, Chiba 263-8522, Japan; ynomura@chiba-u.jp

**Keywords:** lung cancer, endobronchial ultrasonography (EBUS), deep learning, convolutional neural network, multi-scale image, multi-feature fusion

## Abstract

Lung cancer is the leading cause of cancer-related deaths globally and ranks among the most common cancer types. Given its low overall five-year survival rate, early diagnosis and timely treatment are essential to improving patient outcomes. In recent years, advances in computer technology have enabled artificial intelligence to make groundbreaking progress in imaging-based lung cancer diagnosis. The primary aim of this study is to develop a computer-aided diagnosis (CAD) system for lung cancer using endobronchial ultrasonography (EBUS) images and deep learning algorithms to facilitate early detection and improve patient survival rates. We propose $M^{3}\text{-}\mathrm{Net}$, which is a multi-branch framework that integrates multiple features through an attention-based mechanism, enhancing diagnostic performance by providing more comprehensive information for lung cancer assessment. The framework was validated on a dataset of 95 patient cases, including 13 benign and 82 malignant cases. The dataset comprises 1140 EBUS images, with 540 images used for training, and 300 images each for the validation and test sets. The evaluation yielded the following results: accuracy of 0.76, F1-score of 0.75, AUC of 0.83, PPV of 0.80, NPV of 0.75, sensitivity of 0.72, and specificity of 0.80. These findings indicate that the proposed attention-based multi-feature fusion framework holds significant potential in assisting with lung cancer diagnosis.

## 1. Introduction

Lung cancer is the leading cause of cancer-related deaths worldwide [1,2,3]. Most lung cancer deaths occur because the disease is typically diagnosed at an advanced stage [3], by which point the optimal window for treatment has already passed. This delay in detection is a key factor contributing to lung cancer’s exceptionally high mortality rate. Consequently, early diagnosis and screening are critical, playing a pivotal role in improving overall survival rates for patients [4]. Lung cancer is broadly categorized into two types: small-cell lung cancer (SCLC) and non-small-cell lung cancer (NSCLC) [5]. SCLC is characterized by rapid tumor growth, a short doubling time, and early metastasis, making early screening, diagnosis, and treatment crucial for improving prognosis. Non-small-cell lung cancer accounts for approximately 85% of all lung cancer cases. Each type often requires distinct treatment approaches. Traditional diagnostic methods for lung cancer primarily rely on medical imaging, such as magnetic resonance imaging (MRI) [6,7], computed tomography (CT) [8], dual-modality positron emission tomographic (PET)–CT imaging [9], and histopathology [10,11]. However, the differences in imaging techniques mean that each method serves unique diagnostic purposes and requires specific interpretation methods [5]. Clinically, doctors typically diagnose lung cancer based on medical imaging, considering factors such as tumor size and location. However, visual inspection is subjective, and traditional imaging-based diagnoses often lead to missed cases. This can result in delayed treatment, causing patients to miss the optimal treatment window [12].

Commonly used medical imaging techniques for lung cancer screening include CT, MRI, and tissue histopathology images. Studies have shown that low-dose CT [8,13,14] is an effective method for the early diagnosis of lung cancer and can significantly improve patient survival rates. However, its drawbacks include radiation exposure and a relatively high rate of false positives in the diagnostic results [8]. In the diagnosis of brain metastases from NSCLC, MRI has shown a higher diagnostic rate compared to CT [15]. However, this has not translated into improved patient survival rates. This highlights the significant challenges that remain in using CT or MRI for early lung cancer diagnosis. The gold standard for lung cancer diagnosis remains pathological evaluation, where tumor tissue is obtained through invasive procedures like biopsies to determine the tumor’s histology and stage. However, biopsy procedures have limitations and associated risks, making them less ideal for certain patients [16]. For example, tissue biopsy procedures may carry the risk of complications, such as infection, bleeding, or damage to surrounding organs [17].

In recent years, with the rapid advancement of computer technology, cancer diagnostic systems based on artificial intelligence (AI) algorithms have begun to gain prominence in medical image diagnosis. These systems have shown great potential in various areas, such as skin disease diagnosis [18,19], heart disease diagnosis based on ECG [20,21], eye disease diagnosis using fundus images [22], nodule screening from CT images [23,24], and brain tumor diagnosis from MRI [25]. AI has also played a crucial role in lung cancer diagnosis [26], demonstrating significant promise in improving diagnostic accuracy and efficiency.

Raza et al. [5] introduced a CT scan image-based lung cancer classification network called Lung-EffNet, which employs transfer learning for model training. Results indicate that this framework outperforms other classification models in terms of both time complexity and diagnostic performance for lung cancer, achieving strong results even on imbalanced datasets. Teramoto et al. [27] designed a lung cancer diagnostic support system based on a deep convolutional neural network, achieving accuracy comparable to that of pathologists. In their experiments, cytological image data were used to train the network. Due to the large size of the ×40 microscope images, they first cropped the images into 768 × 768 pixel squares, and then downsample them to 256 × 256 pixels. These adjusted cell images were then used for training the deep learning network. Ardila et al. [28] developed an end-to-end CT-based lung cancer diagnostic system with the key advantage of allowing direct input of CT data to both locate lesions and diagnose lung cancer. This end-to-end system first trains a cancer ROI detection model to identify three-dimensional (3D) candidate cancer regions within the CT volume. A 3D CNN-based diagnostic network is then trained to assess these ROI regions for cancer. The system achieved diagnostic performance comparable to that of radiologists, significantly improving the accuracy of lung cancer screening.

In addition to deep learning models, machine learning models are also widely applied in lung cancer detection and classification. Singh et al. [29] proposed a supervised machine learning algorithm for classifying CT scan images. Before training the model, they extracted texture and statistical features from the images, using these features to train the machine learning algorithms. Seven different classifiers were tested, and experiments showed that, compared to the other six algorithms, the multilayer perceptron (MLP) achieved the best performance in lung cancer detection. Radiomics is also a crucial tool in medical image processing, allowing high-throughput feature extraction to uncover detailed information from medical images. This approach provides valuable decision support for medical image analysis by revealing additional insights beyond what is visible to the human eye [30]. Hyun et al. [31] explored the use of PET-based radiomics features combined with machine learning algorithms to classify lung cancer subtypes. Experimental results indicated that PET-based radiomics features can aid clinicians in accurately diagnosing lung cancer subtypes, supporting improved clinical decision-making.

However, classifying lung cancer based on CT, MRI, PET, or PET-CT imaging usually requires additional confirmation through cytology or histology [32]. Endobronchial ultrasound (EBUS) is a relatively new bronchoscopic method that plays a significant role in lung cancer diagnosis and staging. EBUS is particularly effective for guiding biopsies or performing transbronchial needle aspiration, offering a higher diagnostic accuracy compared to traditional bronchoscopy [32]. Given the advantages of endobronchial ultrasound (EBUS), including its minimally invasive nature, real-time imaging, lack of radiation [32,33], and high detection rate, AI-assisted diagnostic systems using EBUS images have been proposed to support physicians in diagnosing lung cancer. Li et al. [34] proposed a multimodal fusion framework based on convex probe endobronchial ultrasound (CP-EBUS) for lung cancer diagnosis. This framework utilizes CP-EBUS images from three modes and constructs a three-branch multi-feature fusion module. Each branch is an improved version of SqueezeNet [35], enhanced with a squeeze-and-excitation (SE) attention [36] mechanism to extract more effective features. Finally, the three different feature sets are fused using a feature weighting approach [37]. Chen et al. [33] proposed a CNN-SVM hybrid framework, where a CNN model is used as a feature extractor to generate image features, which are then used to train an SVM classifier. This approach achieved an accuracy of 85%. Research has shown that AI-based diagnostic systems using EBUS images are highly effective for lung cancer detection. However, in cases of highly imbalanced data, even deep learning algorithms may not yield optimal results. This has been confirmed in our previous studies [38]. According to the experimental results, even with data augmentation, the CNN model attained an accuracy of merely 0.63 on the imbalanced dataset. This outcome suggests that data imbalance hinders the CNN model’s ability to effectively learn and extract pertinent features from the data.

This study proposes a new multi-feature fusion network, called $M^{3}\text{-}\mathrm{Net}$. In the name “$M^{3}\text{-}\mathrm{Net}$”, each “M” represents a key concept in the model: the first “M” stands for the multi-branch framework, the second “M” represents multi-feature fusion, and the third “M” refers to multi-scale images. The main idea behind the network is to construct a multi-branch framework, where each branch uses a different EBUS image. The features from the multiple branches are then fused through a feature fusion module. Finally, a classifier is constructed, and the fused features are fed into the classifier layer to output the predicted results. In summary, the main contributions of this paper are as follows:

- We developed a novel multi-feature fusion framework $M^{3}\text{-}\mathrm{Net}$ for lung cancer diagnosis using EBUS images.
- We propose a method for generating multi-scale EBUS images from a single EBUS image.
- We constructed three attention-based multi-feature fusion modules to enhance the effectiveness of feature fusion.
- To address the impact of data imbalance, we customized the loss function weights, training the model with a weighted loss function to mitigate the influence of data imbalance on the experimental results.

The structure of this paper is organized as follows: Section 2 introduces the materials and methods used, providing a detailed description of data acquisition, data preprocessing, multi-scale image generation, the $M^{3}\text{-}\mathrm{Net}$ framework, and the construction of feature fusion modules. Section 3 presents the experiments and results, detailing the experimental setup, evaluation metrics, and classification results. Section 4 discusses the experimental results. Section 5 presents the conclusions drawn from this study.

## 2. Materials and Methods

This section provides a detailed description of the processes involved in data collection, preprocessing, and dataset construction for training the lung cancer classification model using EBUS images. After data collection, initial preprocessing is applied to the raw data, as they cannot be directly used for model training. Additionally, due to the limited amount of data and significant class imbalance, data imbalance poses a challenge. To address this, we employ both data augmentation techniques and a weighted loss function in model training. The proposed $M^{3}\text{-}\mathrm{Net}$ integrates multiple features within a multi-branch framework, offering more efficiency than single-feature approaches. Details on preprocessing and the $M^{3}\text{-}\mathrm{Net}$ framework will be discussed in the following sections.

### 2.1. Data Acquisition

The EBUS images used in this study were collected at Chiba University Hospital. This study was conducted under the Declaration of Helsinki and approved by the Institutional Ethics Committee of Chiba University Graduate School of Medicine (M10393). The data include cases from two periods, September 2019 to April 2020, and January 2022 to May 2022, involving patients who underwent Endobronchial Ultrasound-guided Transbronchial Lung Biopsy (EBUS-TBLB) examination. The data collection was conducted using a bronchial ultrasound system (EU-M30; Olympus Co., Tokyo, Japan ) equipped with a 20 MHz radial probe (UM-S20-20R; Olympus Co., Tokyo, Japan). Under the guidance of the ultrasound probe, radiologists progressively approached the lesion area, taking sufficient samples for biopsy preparation. The process of the EBUS probe approaching the lesion area was recorded as a MOV video format on the monitor. Each case corresponds to a video file, from which the radiologist removes frames without lesion information, keeping only the relevant frames for model training. Additionally, the label information for each case was determined based on the biopsy results.

### 2.2. Data Preprocessing

After thorough selection and screening by radiologists, a total of 95 lung tumor cases were included in this study. Of these, 13 were benign cases, while the remaining 82 were malignant cases. The EBUS case data used in this study were selected through a rigorous screening process by radiologists. The selection criteria applied during the screening are as follows: First, only cases with clearly visible lesion areas and minimal artifacts were selected. Second, all cases included in this study had prior informed consent from the patients. Lastly, cases without diagnostic results were excluded. For each case, a MOV-format video file was generated, which was reviewed frame by frame by the radiologists. Only the frames containing lesion information were retained. This study focuses on developing a lung cancer CAD system based on individual EBUS images. After obtaining MOV video files, which have been pre-screened by radiologists, we first convert the video into individual EBUS image frames. Since the relevant information about the lesion is typically located around the ultrasound probe in the EBUS images, and the images also contain surrounding text, it is necessary to perform a central crop on the original images. This removes irrelevant noise outside the central lesion area. In our previous research (under review), we proposed a scale line-based image cropping (SLBIC) strategy to achieve this [39]. The SLBIC strategy is inspired by the scale markings present in the original image. Using these markings, we determine the coordinates along the image’s diagonal and use the ultrasound probe as the center for cropping, which addresses variations in central crop sizes in the EBUS images. By identifying the top left and bottom right coordinates for the crop area, we standardize the size of the cropped images. Through the SLBIC cropping strategy, we ultimately obtain four EBUS image blocks of different sizes: 4 × 4 cm (Type 1), 3 × 3 cm (Type 2), 2 × 2 cm (Type 3), and 1 × 1 cm (Type 4).

In addition, this study randomly divided the 95 lung tumor cases into training, validation, and test sets, maintaining the same proportion for each category. Table 1 shows the number of benign and malignant cases included in each set (training, validation, and test sets). Additionally, it describes how many EBUS images were selected from the cases in each data subset.

### 2.3. Multi-Scale Image Generation

In the image preprocessing stage, four different sizes of EBUS images were obtained based on the SLBIC image cropping strategy. Additionally, the previously proposed image transformation method called polar coordinate transformation was introduced [39]. Previous studies have shown that training the model with transformed EBUS images can significantly improve the experimental results, so we decided to adopt this approach. Using this method, the four differently sized EBUS images were converted based on the coordinate system and uniformly resized to 224 × 224 pixels. The transformed images were named polar type 1, polar type 2, polar type 3, and polar type 4, respectively. This resulted in a total of eight EBUS images: four obtained through SLBIC cropping and the other four through the coordinate system-based image conversion method. Using SLBIC cropping, four types of EBUS images were obtained. Based on the multi-scale image generation method, three multi-scale images were generated: MS (multi-scale) 1-2-3, MS 1-2-4, and MS 2-3-4. Additionally, according to the four polar EBUS image types, three multi-scale images were also generated: PMS (polar multi-scale) 1-2-3, PMS 1-2-4, and PMS 2-3-4.

**Figure 1:**
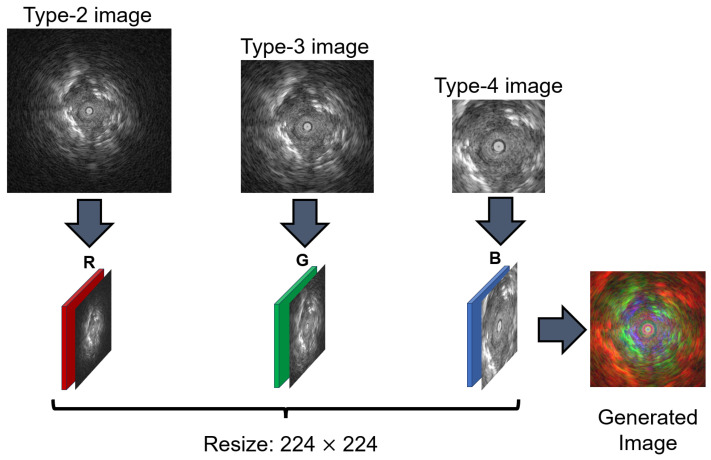
This figure illustrates the detailed process for generating multi-scale EBUS images: First, three EBUS images are selected from the four available types. Each selected EBUS image is then converted from RGB to gray-scale and resized to 224 × 224 pixels. Finally, the three resized gray-scale images are merged to form a single multi-scale RGB image. This approach utilizes varying image resolutions to effectively capture multi-scale features within one composite image.

By placing different EBUS images into the R channel, G channel, and B channel, a composite image containing information from three types of EBUS images is formed. This idea also serves as the inspiration for creating multi-scale images. The process of generating multi-scale images is illustrated in Figure 1. Similar research on generating multi-scale images can be found in the study by Bazargani et al. [40]. In their work, they obtain case images at different resolutions, each corresponding to images of different scales. The detailed process for generating multi-scale EBUS images is as follows: First, three EBUS images are randomly selected from the four available types. Each selected image is then converted from an RGB format to gray-scale and resized to 224 × 224 pixels. Finally, these three single-channel EBUS images are combined into a single multi-scale image by merging them across the RGB channels.

Table 2 presents the combinations of multi-scale images generated using the four types of original EBUS images. Table 3 shows the combinations derived from the EBUS images transformed using the coordinate system-based image conversion method. In this study, six different types of multi-scale images were created: three based on the original EBUS images and another three derived from the coordinate system-based image conversion method.

### 2.4. M^3^-Net: Multi-Feature Fusion Framework

**Figure 2:**
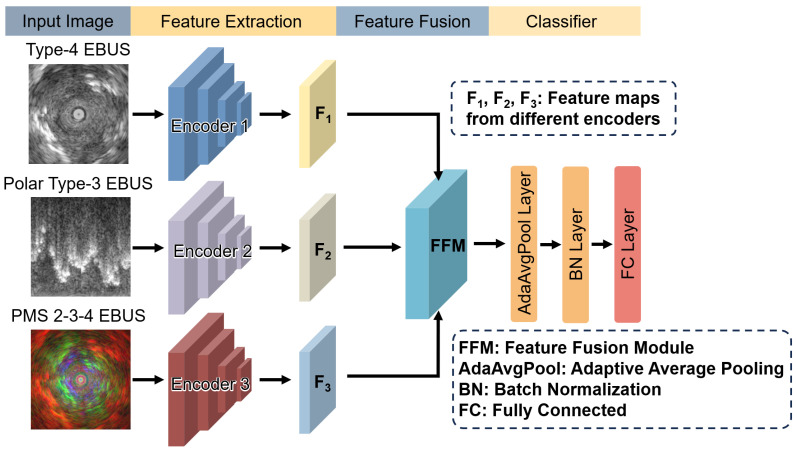
This figure illustrates the architecture of our proposed $M^{3}\text{-}\mathrm{Net}$, which is composed of three key components: a feature extraction module, a feature fusion module, and a classifier. Each branch in the $M^{3}\text{-}\mathrm{Net}$ architecture corresponds to a specific type of EBUS image, with each image type processed by an individual CNN encoder for feature extraction. The extracted features are then sent to the feature fusion module, where they are combined. Finally, the fused features are passed into the classifier to generate the final class prediction. The feature extraction module extracts distinct features, *F*_1_, *F*_2_, and *F*_3_, from different input images. These extracted features are then processed through an attention-based feature fusion module, where they are integrated. Finally, the fused features are passed into the classifier to generate probability predictions for the target categories.

The proposed framework for lung cancer diagnosis is illustrated in Figure 2. Our $M^{3}\text{-}\mathrm{Net}$ model is designed with three primary components. Feature extraction module: $M^{3}\text{-}\mathrm{Net}$ follows a multi-branch architecture, where each branch is dedicated to a specific type of EBUS image. Each EBUS image type is processed by a selected CNN encoder that extracts feature maps (F1, F2, and F3) specific to that image type. The choice of CNN encoder is data-driven, based on experimental results detailed in Section 3.3. Feature fusion module: This module efficiently combines features from the different EBUS images, leveraging an attention mechanism to assign higher weights to features that contain critical information. This enables the model to prioritize the most relevant features. In this study, we experimented with three different fusion module designs, which are described in detail in Section 2.5. Classifier module: After feature fusion, the combined features are sent to the classifier module for the final prediction. The classifier consists of three main layers: an adaptive average pooling layer, which adjusts feature maps for connection to subsequent fully connected layers; a batch normalization layer, which enhances model stability through standardization; and a fully connected layer, which outputs the predicted probabilities.

### 2.5. Feature Fusion Modules

To enhance multi-feature fusion, we designed three distinct attention-based fusion modules, each aimed at effectively integrating multiple feature types. Generally, compared to traditional single-feature CNN models, multi-feature fusion models offer improved classification performance [41], which is the core principle behind the $M^{3}\text{-}\mathrm{Net}$ framework. This approach allows $M^{3}\text{-}\mathrm{Net}$ to leverage complementary information across different feature sets, contributing to a more robust and accurate diagnostic model. Additionally, numerous studies have explored the development of attention-based feature fusion modules [42,43,44,45], as attention mechanisms assign greater weight to key features, enabling the network to focus more effectively on valuable information. Next, the details of each feature fusion module will be described in detail.

**Figure 3:**
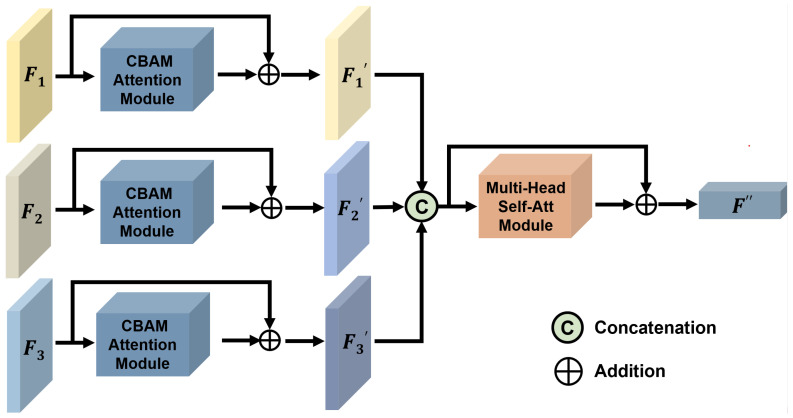
This figure illustrates the framework of feature fusion module version 1 (FFM-v1).

The specifics of feature fusion module version 1 (FFM-v1) are shown in Figure 3. Here, $F_{1}$, $F_{2}$, and $F_{3}$ represent feature maps extracted from different encoders. Each of these feature maps is then processed through a CBAM (Convolutional Block Attention Module) attention [46] layer, which assigns weights to the feature maps based on their significance. A residual connection [47] is incorporated into the attention module for two main reasons: to mitigate the gradient vanishing problem as the network depth increases and to ensure that information from previous layers is effectively transferred to subsequent layers without loss due to the introduction of attention. After weighting, we obtain feature maps $F_{1}^{\prime }$, $F_{2}^{\prime }$, and $F_{3}^{\prime }$. These are then concatenated into a unified feature map. This concatenated feature map is subsequently fed into a multi-head self-attention module [48], selected for its ability to allow the model to focus on information from multiple subspaces at various positions simultaneously. The final output is the fused feature map $F^{\prime \prime }$.

**Figure 4:**
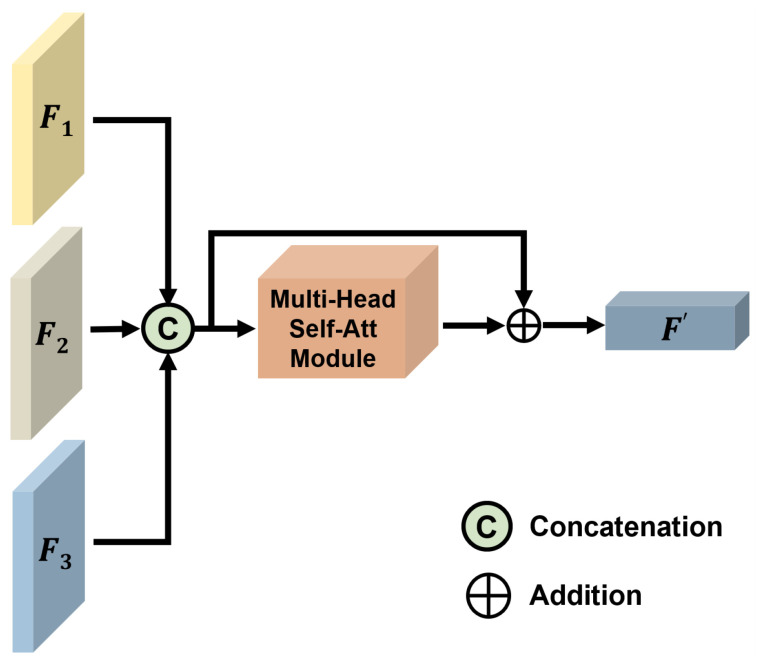
This figure illustrates the framework of feature fusion module version 2 (FFM-v2).

The details of feature fusion module version 2 (FFM-v2) are illustrated in Figure 4. FFM-v2 is the simplest among the three fusion modules. It builds upon the concatenation of features from different branches by incorporating a multi-head self-attention mechanism. Feature maps $F_{1}$, $F_{2}$, and $F_{3}$ are extracted from different encoders and concatenated. This concatenated feature map is then used as the input to the multi-head self-attention mechanism, where attention weights are applied. Finally, a residual connection merges the pre-attention input feature map with the post-attention feature map through an addition operation, resulting in the final feature map $F^{\prime \prime }$.

**Figure 5:**
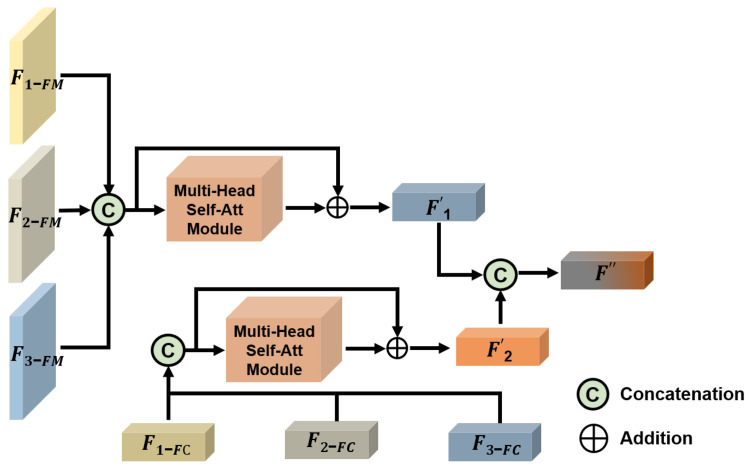
This figure illustrates the framework of feature fusion module version 3 (FFM-v3).

The details of feature fusion module version 3 (FFM-v3) are shown in Figure 5. The concept behind FFM-v3 is to effectively integrate feature maps with fully connected (FC) layer features. Feature maps generally retain local spatial information of the image, while FC layer features represent global information. This fusion enables the model to capture both local and global image features, enhancing overall performance. The detailed process is as follows: $F_{1\text{-}\mathrm{FM}}$ and $F_{1\text{-}\mathrm{FL}}$ are derived from Encoder 1, $F_{2\text{-}\mathrm{FM}}$ and $F_{2\text{-}\mathrm{FL}}$ from Encoder 2, and $F_{3\text{-}\mathrm{FM}}$ and $F_{3\text{-}\mathrm{FL}}$ from Encoder 3. Here, FM denotes feature map features, and FC denotes fully connected layer features. The handling of both types of features is generally similar. First, the features are concatenated and fed into a multi-head self-attention module. Then, a residual connection merges the concatenated features with the output from the attention module. Finally, another concatenation operation fuses the processed feature map features $F_{1}^{\prime }$ with the processed FC layer features $F_{2}^{\prime }$ after attention processing.

### 2.6. EBUS Image Encoder Architecture

**Figure 6:**
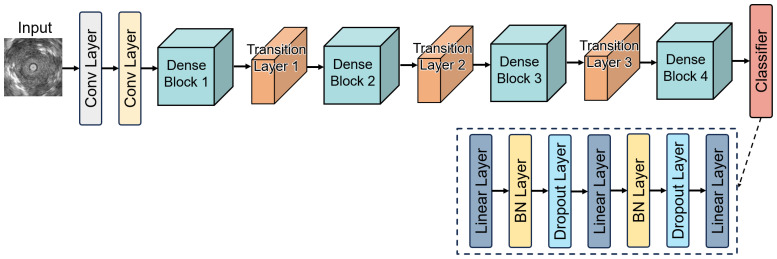
This figure represents the encoder framework based on the fine-tuned DenseNet-121 model.

The encoders used for EBUS image feature extraction are built upon the concept of transfer learning [49] and are fine-tuned based on several commonly used CNN models. This approach leverages pre-trained models to adapt their knowledge for the specific task of EBUS image analysis. By fine-tuning the classifier of these models, the framework effectively repurposes them for feature extraction in this specialized domain. This study utilized 12 CNN models pre-trained on the ImageNet dataset [50]: ResNet-18, ResNet-50, ResNet-101 [47], ResNext-50, ResNext-101 [51], DenseNet-121, DenseNet-169 [52], DenseNet-201, MobileNet-V2 [53], ShuffleNet-V2 [54], EfficientNet-B0 [55], and EfficientNet-V2 [56]. For each CNN model, only the final classifier was replaced. The details of the CNN encoder (DenseNet-121) are shown in Figure 6. The new classifier consisted of three fully connected layers, with batch normalization and Dropout layers added after every two fully connected layers. These additions served to accelerate model convergence and prevent overfitting, respectively. Before being used as encoders for feature extraction, the CNN models underwent fine-tuning on our dataset. The initial weights for each CNN model were sourced from their pre-trained weights on the ImageNet dataset. After fine-tuning, the CNN models were frozen, meaning their weights remained unchanged during feature extraction. This ensured stable and consistent feature extraction for subsequent stages of the framework.

### 2.7. Weight Definition for Loss Function

In the field of medical image diagnosis, data imbalance is particularly common. When a dataset is highly imbalanced, the classification performance of algorithms can be significantly affected [57]. Moreover, as the degree of imbalance increases, the performance loss escalates rapidly [58]. Our study also encountered severe data imbalance, as detailed in Table 1. The dataset contained approximately 6 to 7 times more malignant cases than benign ones. The cause of this situation lies in the fact that malignant lung tumors typically present with obvious clinical symptoms, requiring aggressive treatment. During the treatment process, patients undergo numerous tests, diagnoses, and treatments, resulting in the generation of extensive clinical data. In contrast, many patients with benign lung tumors experience mild or even no symptoms, and their condition is often discovered incidentally during routine check-ups or other examinations. As a result, these patients tend to seek medical attention and undergo diagnosis less frequently, as their benign tumors do not show significant symptoms. To mitigate the impact of this imbalance, we introduced a weighted loss function to compensate for the training loss bias of minority classes. Additionally, when constructing the dataset, we ensured that the training, validation, and test sets contained an equal number of images from each class as much as possible. This approach and the weighted loss function during CNN model training further balanced the distributional bias. The weight calculation process is as follows: (1) Determine the number of benign and malignant cases, $N_{b}$ and $N_{m}$, in the training set during model training. (2) Normalize these counts to obtain $N_{b_{norm}}$ and $N_{m_{norm}}$. (3) Compute their reciprocals, ensuring that the weights are inversely proportional to the number of cases: $\frac{1}{N_{b_{norm}}}$ and $\frac{1}{N_{m_{norm}}}$. (4) Normalize these reciprocals again to obtain the final weights used for training.

## 3. Experiment and Results

This section provides a detailed discussion of the experimental setup, including the specifications of the experimental platform, implementation details of the model, hyperparameter settings, definitions of evaluation metrics, and a presentation of the experimental results.

### 3.1. Experimental Setting

This study was conducted using the PyTorch 2.0.1 deep learning framework, with all experiments running on a workstation with an Intel Core i5-13600K 3.50 GHz six-core processor (Intel Corporation, Santa Clara, CA, USA) and NVIDIA TITAN graphics processing unit (GPU) (NVIDIA Corporation, Santa Clara, CA, USA) equipped with 24 GB of GPU memory. We utilized the Adam optimizer with weight decay, setting the decay coefficient to $5\times 10^{-4}$. The network was trained for 200 epochs, with a batch size of 32, and input images were uniformly resized to 224 × 224 pixels. We used weighted cross-entropy as the loss function and set the initial learning rate to $1\times 10^{-3}$. To prevent overfitting, early stopping was applied, with a patience parameter of six epochs. During training, data augmentation techniques were employed, including horizontal and vertical flips, 90-degree rotations, and random adjustments to brightness, contrast, and saturation. Additionally, all images were normalized before input, scaling pixel values from [0, 255] to the range [0, 1]. The hyperparameters were determined through ablation studies, with the selected configuration shown to yield optimal performance.

The implementation details of the $M^{3}\text{-}\mathrm{Net}$ framework are as follows. First, a two-stage training strategy is adopted. In the first stage, CNN models are trained separately on different EBUS image modalities to obtain CNN encoders for feature extraction. In the second stage, the trained CNN models are used as feature extractors. It is important to note that during feature extraction, the weights of each encoder remain frozen and are not updated. When training the feature fusion module and classifier, the CNN encoders do not participate in further training.

### 3.2. Evaluation Metrics

Seven metrics were used to evaluate the performance of the lung cancer diagnostic system: Accuracy, F1-score, Area Under the Curve (AUC), Positive Predictive Value (PPV), Negative Predictive Value (NPV), sensitivity (SEN), and specificity (SPEC). The definitions of these evaluation metrics are as follows: (1)–(6). In addition, this study employed four-fold cross-validation to ensure that the model evaluation results were more reliable and rigorous.

(1) $$Accuracy(Acc)=\frac{TP+TN}{TP+TN+FP+FN}$$

(2) $$F1-Score=\frac{2\times \mathrm{Precision}\times \mathrm{Recall}}{\mathrm{Precision}\;+\;\mathrm{Recall}}$$

(3) $$Positive\;Predictive\;Value(PPV)=\frac{TP}{TP+FP}$$

(4) $$Negative\;Predictive\;Value(NPV)=\frac{TN}{TN+FN}$$

(5) $$Sensitivity(Sen)=\frac{TP}{TP+FN}$$

(6) $$Specificity(Spec)=\frac{TN}{TN+FP}$$

Additionally, among the seven evaluation metrics mentioned above, AUC is a comprehensive measure derived from the ROC (receiver operating characteristic) curve. The other metrics are computed at a specific threshold. In this study, AUC is considered the most important evaluation metric. Particularly when dealing with class imbalance or when different evaluation metrics need to be balanced, AUC provides a more objective model evaluation. Therefore, AUC is prioritized in this study to comprehensively assess the model’s overall performance across various classification thresholds, avoiding potential bias that could arise from using a single threshold. This approach offers a more global performance evaluation.

### 3.3. Classification Results

The experiments in this study are divided into two main parts. The first part involves training a CNN-based encoder for feature extraction from EBUS images. The results confirm the effectiveness of the weighted cross-entropy loss function. Subsequently, one optimal EBUS image type is selected from each of the four major categories: (1) four types of EBUS images obtained after cropping, (2) four types of EBUS images after coordinate system transformation, (3) three MS-EBUS (multi-scale) images generated from cropped EBUS images, and (4) three PMS-EBUS (polar multi-scale) images generated from transformed EBUS images. Although it is technically feasible to use all 14 types of EBUS images, this approach would create numerous combinations and significantly increase the time complexity. The primary aim of this study is to integrate different EBUS image types. Thus, based on experimental results, the most effective image from each category is chosen for feature fusion, ensuring both feature diversity and reduced experimental complexity. In the second part of the experiment, the four selected EBUS image types are used with the trained CNN model as the feature extraction encoder within the proposed $M^{3}\text{-}\mathrm{Net}$ framework. The experimental results confirm the effectiveness of this framework.

**Figure 7:**
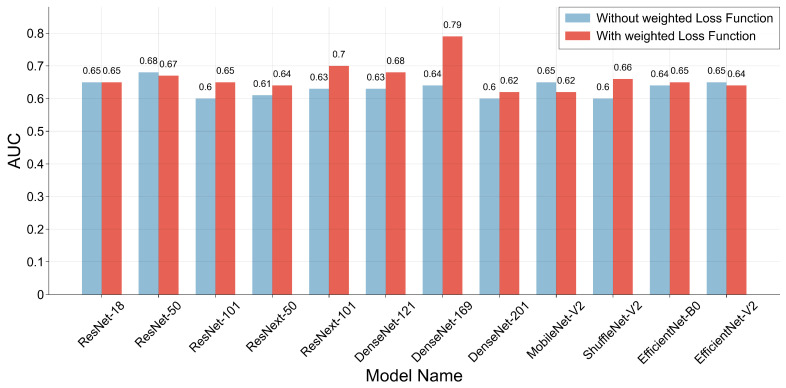
This figure illustrates 12 CNN models that were trained on the polar type 3 EBUS dataset using both unweighted cross-entropy and weighted cross-entropy as loss functions to evaluate their AUC.

In the first part of the experiment, 12 different CNN models were tested on the polar type 3 EBUS dataset, with all results reported as cross-validation averages. The models were trained using both unweighted cross-entropy and weighted cross-entropy as loss functions. As shown in Figure 7, the results indicate that over 70% of the CNN models showed performance improvements when trained with the weighted cross-entropy. Notably, the best performance was achieved with the DenseNet-169 model, reaching an AUC of 0.79.

**Figure 8:**
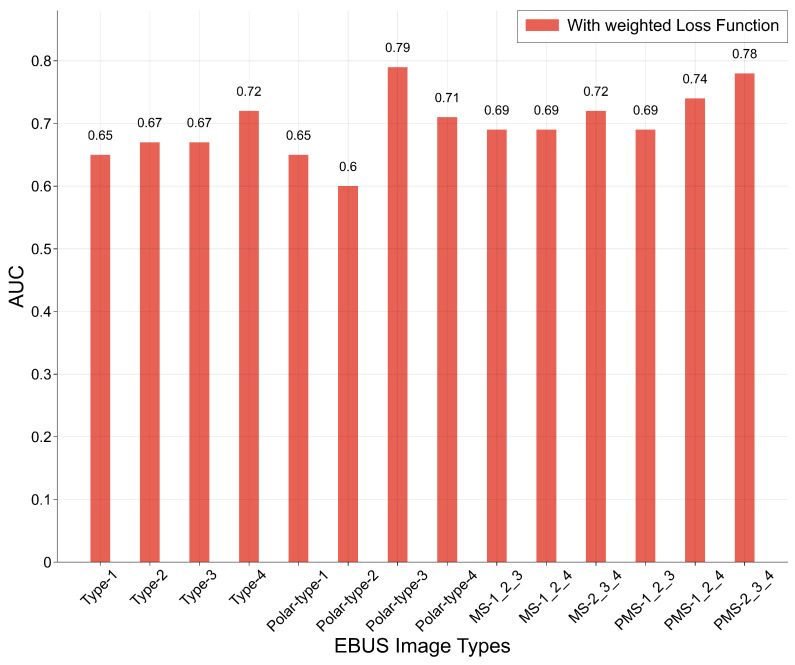
This figure shows the best AUC achieved when training 12 CNN models with weighted cross-entropy loss on each of the 14 EBUS image datasets.

According to the experimental results shown in Figure 7, training CNN models with weighted cross-entropy effectively enhances performance. Currently, there are four major categories of EBUS image datasets, comprising a total of 14 types of EBUS images. From each category, the most effective EBUS image dataset will be selected for training within the $M^{3}\text{-}\mathrm{Net}$ framework. Using weighted cross-entropy as the loss function, 12 CNN models were trained on each of the 14 EBUS image datasets. The best results for each dataset are presented in Figure 8, with all results representing cross-validation averages. For the detailed performance of the best-performing models in each image type and specific information on the selected CNN models, please refer to the appendix in Appendix A. Based on the experimental results shown in Figure 8, the highest AUC of 0.79 was achieved on the polar type 3 EBUS image datasets. Additionally, within each of the four major EBUS image categories, the best-performing datasets were type 4, polar type 3, MS 2-3-4, and PMS 2-3-4, with an AUC of 0.72, 0.79, 0.72, and 0.78, respectively. Based on the experimental results, the four selected EBUS image types from the major categories are type 4, polar type 3, MS 2-3-4, and PMS 2-3-4. Details of the CNN models used to achieve the highest AUC for each dataset, along with other evaluation metrics, are provided in Table 4.

Based on the experimental results, the most effective EBUS images from each category and their corresponding CNN model were selected as encoders for feature extraction. These were then used in the proposed $M^{3}\text{-}\mathrm{Net}$ framework for multi-feature fusion. As shown in Table 4, four types of EBUS images were selected for the multi-feature fusion stage within the proposed $M^{3}\text{-}\mathrm{Net}$ framework. During this stage, there are multiple ways to combine these four image types. The fusion options include the following: (1) combining two types of EBUS images, (2) combining three types, and (3) combining all four types. Only a selection of representative experimental results is presented here. All experimental results are the final averages obtained through four-fold cross-validation.

The experimental results for feature fusion using the proposed $M^{3}\text{-}\mathrm{Net}$ multi-feature fusion framework, combining type 4 and polar type 3 features, are shown in Table 5. The results showed varying degrees of improvement when using three different fusion modules. Specifically, with FFM-v2, the accuracy improved by up to 2%, and the AUC increased by up to 4% compared to using the single modality polar type 3.

Table 6 presents the experimental results of the proposed $M^{3}\text{-}\mathrm{Net}$ multi-feature fusion framework, integrating features from polar type 3 and multi-scale PMS 2-3-4 images. The results show that using the three different fusion modules led to varying degrees of improvement. Specifically, with FFM-v3, the AUC improved by up to 3%, compared to the single-modality polar type 3.

Table 7 presents the experimental results of the proposed $M^{3}\text{-}\mathrm{Net}$ multi-feature fusion framework, integrating features from type 4, polar type 3, and PMS 2-3-4 images. The results show that using FFM-v3 increased the AUC by 8% compared to the single-modality type 4 and by 1% compared to the single-modality polar type 3.

Table 8 presents the experimental results of the proposed $M^{3}\text{-}\mathrm{Net}$ multi-feature fusion framework, combining features from type 4, polar type 3, MS 2-3-4, and PMS 2-3-4 images. The results show that using FFM-v3 improved the AUC by 5% compared to the single-modality type 4 and MS 2-3-4. However, the AUC decreased by 2% and 1% compared to the single-modalities polar type 3 and PMS 2-3-4, respectively.

Table 9 presents the comparison of experimental results between the proposed $M^{3}\text{-}\mathrm{Net}$ multi-feature fusion framework and previously published methods. Due to the unavailability of data from related studies, we replicated the experiments using our own dataset, strictly following the procedures described in the original papers. Cross-validation was employed in all experiments. According to the experimental results, our proposed $M^{3}\text{-}\mathrm{Net}$ shows an improvement of approximately 9% in accuracy and 30% in AUC compared to the previously published methods. Compared to our previous research, the accuracy increased by about 10%, and the AUC improved by more than 11%.

## 4. Discussion

$M^{3}\text{-}\mathrm{Net}$ is designed to integrate features from multiple types of images. It first trains separate CNN models on different EBUS image types, which are then used as feature encoders. These encoders extract features from various EBUS images, which are subsequently fused through an attention-based feature fusion module. The final output is the prediction result. Experimental results after feature fusion demonstrate that the proposed $M^{3}\text{-}\mathrm{Net}$ multi-feature fusion framework effectively improves lung cancer diagnostic performance.

In our study, a total of 95 cases were included, comprising 13 benign and 82 malignant cases, with each case corresponding to an EBUS video. During dataset preparation, unlike other studies [33,59] that typically select a single EBUS image per case, we opted not to follow this approach. Selecting only one image per case would have resulted in an extremely imbalanced and small dataset, which could lead to poor experimental results. In our study, we selected EBUS images evenly from each case video based on its length, ensuring that the numbers of benign and malignant EBUS images included in the training, validation, and test sets were balanced, as shown in Table 1. While the number of EBUS images for each class was equal, there was a significant disparity in the number of cases within each class. This discrepancy motivated our proposal to define custom weights for the loss function based on the number of cases in the dataset. By training CNN models using a weighted loss function, as illustrated in Figure 7, the experimental results show that this approach can further improve the accuracy of CNN models. Coupled with ensuring an equal number of EBUS images per class in the dataset, this strategy enhances data balance. According to the results in Table 4, CNN models trained with the weighted loss function on single-modal data, such as polar type 3 and MS 2-3-4 EBUS images, achieved similar sensitivity and specificity. If CNN models are trained on highly imbalanced datasets without any balancing strategy, the resulting models often exhibit significantly higher sensitivity than specificity. This further confirms the effectiveness of the weight definition for the loss function strategy in ensuring data balance and improving model performance.

Table 5 and Table 6 present the experimental results of integrating two types of EBUS image features using the proposed $M^{3}\text{-}\mathrm{Net}$ multi-feature fusion framework. The results demonstrate that employing any of the three feature fusion modules yields notable performance improvements. Furthermore, the results indicate that the sensitivity and specificity achieved through $M^{3}\text{-}\mathrm{Net}$ are remarkably close, suggesting that the proposed framework effectively mitigates the impact of imbalanced data. As shown in Table 5, the sensitivity observed for type 4 EBUS images in single-modality experiments is unexpectedly lower than the specificity. These results were obtained through four-fold cross-validation, with the final outcome calculated as the average across all folds. Although sensitivity may exceed specificity in some folds, the averaged sensitivity remains 22% lower than specificity. This discrepancy could be attributed to over-correction during model training caused by the weighted loss function.

Similarly, Table 6 reveals a comparable issue in single-modality experiments conducted on PMS 2-3-4 EBUS images, where sensitivity is 15% lower than specificity. This discrepancy might be due to the limited number of cases in the dataset, leading to distribution imbalances among training, validation, and test sets in each fold. To address these issues, future efforts will focus on collecting more data to enhance the framework’s robustness and generalization capabilities. Additionally, a dynamic weighting strategy for the loss function will be explored, replacing the current method, which relies solely on the proportion of benign and malignant cases in the training set to determine the weight.

Table 7 presents the experimental results of integrating three EBUS image features using the proposed $M^{3}\text{-}\mathrm{Net}$ multi-feature fusion framework. The results show that the performance improvement is limited in some cases. For instance, when using the FFM-v3 fusion module, accuracy and AUC improved by only 1% compared to the single-modality polar type 3 results, which deviates from expectations. When integrating type 4 and polar type 3 EBUS images through the $M^{3}\text{-}\mathrm{Net}$ framework, the accuracy and AUC achieved maximum improvements of 3% and 4%, respectively, compared to single-modality polar type 3. Similarly, when fusing polar type 3 and PMS 2-3-4 EBUS images, the accuracy and AUC improvements were 1% and 3%, respectively. However, when combining features from type 4, polar type 3, and PMS 2-3-4, the accuracy and AUC showed only a 1% improvement, which falls short of expectations. Theoretically, integrating multiple features should enhance the information available for determining class boundaries, leading to improved results. However, during feature fusion, hidden information loss may occur [60], as feature extraction for each component is performed independently before fusion. This loss could explain the limited performance gains observed in the experiments. This phenomenon may also explain why the performance decreases rather than improves when we use $M^{3}\text{-}\mathrm{Net}$ to fuse four types of EBUS image features. For detailed results on fusing four types EBUS image feature, please refer to Table 8.

To assess the contribution of individual components to model performance, we conducted an ablation study evaluating the impact of the weighted loss function, feature fusion module, and data augmentation. The results are summarized in Table 10. When none of the components were included, the model achieved an AUC of 0.74 and an accuracy of 0.68, serving as the baseline performance. Introducing the weighted loss function alone resulted in a slight decrease in AUC to 0.72, while accuracy remained at 0.68. This suggests that the weighted loss function may not directly improve AUC when used in isolation, but could have a positive impact when combined with other techniques. Incorporating the feature fusion module alone increased the AUC to 0.76 and accuracy to 0.71, indicating that feature fusion effectively integrates complementary information, thereby enhancing model performance. When only data augmentation was applied, the AUC remained at 0.74, but accuracy dropped to 0.64, suggesting that data augmentation alone introduced greater variability without yielding a significant performance improvement. When all three components were incorporated, the model achieved the highest AUC (0.83) and accuracy (0.76), confirming the complementary nature of these techniques. This further demonstrates that synergy among multiple strategies effectively enhances overall performance.

The specific details of the feature fusion module (FFM-v1) are illustrated in Figure 3. Features $F_{1}$, $F_{2}$, and $F_{3}$, represent the feature maps extracted from different encoders. After being processed by the CBAM attention layer, these features are concatenated and fed into a multi-head self-attention module. In contrast, the FFM-v2 is simpler: it concatenates the features from different encoders and directly feeds them into the multi-head self-attention module. Compared to FFM-v1, the early fusion of features in FFM-v2 is more effective. The feature maps after the CBAM attention module may alter the model’s representation, and using two different attention mechanisms can increase the training complexity of the model. This could be one reason why the experimental results of FFM-v1 are not as good as those of FFM-v2.

The feature fusion module FFM-v3 builds on FFM-v2 by adding additional fully connected layers. While the intention was to provide the model with more diagnostic information, the experimental results indicate that FFM-v3 performs worse than FFM-v2. This suggests that, although adding extra information through the fully connected layers might seem beneficial, it could also introduce noise, which in turn reduces model performance. Although our proposed multi-feature fusion framework, $M^{3}\text{-}\mathrm{Net}$, achieves satisfactory improvements compared to single-modality approaches, it has certain limitations. This study utilized over ten CNN models as feature extractors for EBUS images. CNNs primarily rely on local operations through convolutional filters. While convolution is advantageous for developing efficient algorithms, its limited receptive field restricts the ability to capture long-range pixel relationships [61]. In recent years, Transformer-based techniques have gained increasing attention in the medical imaging domain, especially with the advent of ViT (Vision Transformer) [62]. This is largely because Transformers can effectively capture global contextual information. Considering these advantages, future research will explore using ViT-based models for feature extraction. Secondly, our study revealed that as the number of EBUS image modalities increased, the fusion results unexpectedly deteriorated. This could be attributed to misalignment in high-dimensional feature spaces. While CNN encoders extract more effective features from different EBUS image types, these features may lack alignment, and simple concatenation fails to address this issue. To resolve this, future research may explore the use of adversarially regularized encoders [63] to project features extracted from different EBUS images into a shared feature space. By aligning these features, we can train the classifier more effectively and improve the fusion results.

Additionally, this study lacks ablation experiments to verify the contribution of multi-scale fusion to the improvement of the framework’s classification performance. In future research, we will conduct ablation experiments to further demonstrate the effectiveness of multi-scale fusion. The amount of data included in the experiments is indeed insufficient, and external validation has not been conducted. In our future research, we will first collect more data and then perform external validation as well as multi-center validation to assess the model’s performance and generalization ability, ensuring its applicability in clinical settings. In future research, we plan to incorporate the gradient-weighted class activation mapping (Grad-CAM) method [64] to analyze the model’s decision-making process by visualizing key areas in the images that contribute significantly to the predictions of malignancy or benignancy. Additionally, we will use Shapley additive explanation (SHAP) values [65] to quantify the influence of individual features on the model’s output. This will enable us to gain a clearer understanding of how the model makes predictions and ensure that its decisions are aligned with clinical reasoning.

## 5. Conclusions

This study proposes a multi-scale image generation method and, based on the generated multi-scale images, constructs the $M^{3}\text{-}\mathrm{Net}$ CAD framework for lung cancer diagnosis. To address the impact of data imbalance, the weight of the loss function was customized, and a weighted cross-entropy loss function was used to train the CNN model, alleviating the effects of the imbalance. The trained CNN model was then used as the feature extractor in the $M^{3}\text{-}\mathrm{Net}$ multi-feature fusion framework, with the weights of the CNN model frozen during the training of the $M^{3}\text{-}\mathrm{Net}$ multi-feature fusion framework. Additionally, three attention-based feature fusion modules were introduced to enhance the effectiveness of feature fusion. The experimental results show that FFM-v2 achieved the best performance, with an accuracy of 0.76, F1-score of 0.75, AUC of 0.83, PPV of 0.80, NPV of 0.75, sensitivity of 0.72, and specificity of 0.80.

In conclusion, the deep learning-based computer-aided diagnostic system for lung cancer diagnosis has shown promising results. Although the dataset used in this study suffers from an imbalance issue, future work will include not only the use of data augmentation techniques but also the collection of more data to further validate the robustness of the $M^{3}\text{-}\mathrm{Net}$ multi-feature fusion framework.

## Figures and Tables

**Table 1:** The table shows the dataset division, detailing how many benign and malignant cases were included in each subset. It also indicates the number of EBUS images selected from the included cases.

	Benign		Malignant	
	**Image Nums**	**Case Nums**	**Image Nums**	**Case Nums**
Train	270	6	270	40
Valid	150	3	150	21
Test	150	4	150	21
Overall	570	13	570	82

**Table 2:** This table displays the various combinations of multi-scale (MS) images generated using the four types of EBUS images. Each combination represents different sets of three EBUS images selected from the four available types, merged into a single RGB image to capture multi-scale features from diverse resolutions. This approach aims to enhance the model’s ability to detect patterns across scales, improving classification effectiveness.

Generated Multi-Scale Image	Type 1	Type 2	Type 3	Type 4
MS 1-2-3	Used	Used	Used	**-**
MS 1-2-4	Used	Used	**-**	Used
MS 2-3-4	**-**	Used	Used	Used

**Table 3:** This table presents the polar multi-scale (PMS) image combinations generated from four types of EBUS images using the coordinate system-based image conversion method. PMS stands for polar multi-scale. Each combination represents a selection of three different EBUS image types from the four available, which are then merged into a single RGB image. This configuration is designed to capture multi-scale features across various resolutions, enhancing the model’s ability to recognize patterns across scales and thus improving classification performance.

Generated Multi-Scale Image	Polar Type 1	Polar Type 2	Polar Type 3	Polar Type 4
PMS 1-2-3	Used	Used	Used	**-**
PMS 1-2-4	Used	Used	**-**	Used
PMS 2-3-4	**-**	Used	Used	Used

**Table 4:** This table shows the best results achieved on the selected four types of EBUS image datasets—type 4, polar type 3, MS 2-3-4, and PMS 2-3-4. The details of these networks and evaluation metrics other than accuracy are summarized. The experimental result of each model is the average result after the cross-validation.

Image Type	CNN Model	AUC	Acc	F1-Score	PPV	NPV	Sen	Spec
Type 4	DenseNet-121	0.72	0.70	0.66	0.77	0.67	0.59	0.81
Polar Type 3	DenseNet-169	0.79	0.74	0.72	0.79	0.74	0.70	0.77
MS 2-3-4	DenseNet-201	0.72	0.71	0.70	0.71	0.73	0.71	0.72
PMS 2-3-4	MobileNet-V2	0.78	0.74	0.72	0.79	0.70	0.66	0.81

**Table 5:** This table shows the experimental results of the fusion of type 4 and polar type 3 based on the $M^{3}\text{-}\mathrm{Net}$ framework and the comparison result with the single modality.

	Image Type	CNN Encoder	Feature Fusion Module	AUC	Acc	F1-Score	PPV	NPV	Sen	Spec
Single Model	Type 4	DenseNet-121	**-**	0.72	0.70	0.66	0.77	0.67	0.59	0.81
	Polar Type 3	DenseNet-169	**-**	0.79	0.74	0.74	0.79	0.74	0.70	0.77
$M^{3}\text{-}\mathrm{Net}$ Framework	Type 4+ Polar Type 3	DenseNet-121+ DenseNet-169	FFM-v1	0.81	0.75	0.72	0.80	0.74	0.70	0.79
			FFM-v2	0.83	0.76	0.75	0.80	0.75	0.72	0.80
			FFM-v3	0.82	0.77	0.76	0.79	0.78	0.76	0.77

**Table 6:** This table shows the experimental results of the fusion of polar type 3 and PMS 2-3-4 based on the $M^{3}\text{-}\mathrm{Net}$ framework and comparison result with the single modality.

	EBUS Image Type	CNN Encoder	Feature Fusion Module	AUC	Acc	F1-Score	PPV	NPV	Sen	Spec
Single Model	Polar Type 3	DenseNet-169	**-**	0.79	0.74	0.74	0.79	0.74	0.70	0.77
	PMS 2-3-4	MobileNet-V2	**-**	0.78	0.74	0.72	0.79	0.70	0.66	0.81
$M^{3}\text{-}\mathrm{Net}$ Framework	Polar Type 3+ PMS 2-3-4	DenseNet-169+ MobileNet-V2	FFM-v1	0.80	0.75	0.74	0.77	0.74	0.72	0.78
			FFM-v2	0.80	0.74	0.75	0.74	0.75	0.76	0.72
			FFM-v3	0.82	0.75	0.75	0.78	0.74	0.72	0.79

**Table 7:** This table shows the experimental results of the fusion of type 4, polar type 3, and PMS 2-3-4 based on the $M^{3}\text{-}\mathrm{Net}$ framework and the comparison result with the single modality.

	EBUS Image Type	CNN Encoder	Feature Fusion Module	AUC	Acc	F1-Score	PPV	NPV	Sen	Spec
Single Model	Type 4	DenseNet-121	**-**	0.72	0.70	0.66	0.77	0.67	0.59	0.81
	Polar Type 3	DenseNet-169	**-**	0.79	0.74	0.74	0.79	0.74	0.70	0.77
	PMS 2-3-4	MobileNet-V2	**-**	0.78	0.74	0.72	0.79	0.70	0.66	0.81
$M^{3}\text{-}\mathrm{Net}$ Framework	Type 4+ Polar Type 3+ PMS 2-3-4	DenseNet-121+ DenseNet-169+ MobileNet-V2	FFM-v1	0.78	0.74	0.74	0.76	0.73	0.72	0.76
			FFM-v2	0.76	0.72	0.73	0.73	0.75	0.75	0.70
			FFM-v3	0.80	0.75	0.74	0.77	0.75	0.73	0.78

**Table 8:** This table shows the experimental results of fusing four different EBUS images based on the $M^{3}\text{-}\mathrm{Net}$ framework, compared with the results using a single modality.

	EBUS Image Type	CNN Encoder	Feature Fusion Module	AUC	Acc	F1- Score	PPV	NPV	Sen	Spec
Single Model	Type 4	DenseNet-121	**-**	0.72	0.70	0.66	0.77	0.67	0.59	0.81
	Polar Type 3	DenseNet-169	**-**	0.79	0.74	0.74	0.79	0.74	0.70	0.77
	MS 2-3-4	DenseNet-201	**-**	0.72	0.71	0.70	0.71	0.73	0.71	0.72
	PMS 2-3-4	MobileNet-V2	**-**	0.78	0.74	0.72	0.79	0.70	0.66	0.81
$M^{3}\text{-}\mathrm{Net}$ Framework	Type 4+Polar Type 3+MS 2-3-4 +PMS 2-3-4	DenseNet-121+Dense Net-169+DenseNet -201+MobileNet-V2	FFM-v1	0.72	0.68	0.70	0.66	0.73	0.77	0.59
			FFM-v2	0.75	0.71	0.73	0.69	0.74	0.77	0.66
			FFM-v3	0.77	0.73	0.73	0.73	0.73	0.74	0.71

**Table 9:** This table presents a comparative analysis of the experimental results between the proposed $M^{3}\text{-}\mathrm{Net}$ multi-feature fusion framework and existing published methods.

	Methods	AUC	Acc	F1-Score	PPV	NPV	Sen	Spec
Published methods	CNN-SVM [33]	0.54	0.68	0.80	0.88	0.14	0.74	0.33
	Single image-based model [59]	0.52	0.67	0.79	0.87	0.12	0.73	0.31
Previous work [38]	Majority Voting	0.66	0.66	0.59	0.76	0.62	0.52	0.79
	Output Average	0.67	0.67	0.62	0.75	0.64	0.56	0.77
	Performance Weighting	0.72	0.66	0.60	0.76	0.62	0.53	0.78
Our proposed	$M^{3}\text{-}\mathrm{Net}$	0.83	0.76	0.75	0.80	0.75	0.72	0.80

**Table 10:** Ablation study results evaluating the impact of the weighted loss function, feature fusion module, and data augmentation on model performance. The AUC and Acc are reported for each setting.

Ablation Setting			AUC	Acc
Weighted Loss Function	Feature Fusion Module	Data Augmentation		
✗	✗	✗	0.74	0.68
✓	✗	✗	0.72	0.68
✗	✓	✗	0.76	0.71
✗	✗	✓	0.74	0.64
✓	✓	✓	0.83	0.76

## Data Availability

Data are contained within the article.

## Appendix A

**Table A1:** This table presents the detailed results of the best-performing models for each type of EBUS image, along with the specific CNN backbone networks selected.

	Image Type	CNN Model	AUC	Acc	F1-Score	PPV	NPV	Sen	Spec
Original EBUS data	Type 1	ResNet-50	0.65	0.65	0.55	0.81	0.61	0.46	0.84
	Type 2	ResNet-50	0.67	0.67	0.61	0.74	0.63	0.53	0.81
	Type 3	ResNet-18	0.67	0.67	0.60	0.75	0.63	0.51	0.83
	Type 4	DenseNet-121	0.72	0.70	0.66	0.77	0.67	0.59	0.81
Polar EBUS data	Polar Type 1	ResNet-50	0.65	0.65	0.47	0.96	0.60	0.33	0.98
	Polar Type 2	ResNext-101	0.60	0.60	0.38	0.89	0.57	0.29	0.91
	Polar Type 3	DenseNet-169	0.79	0.74	0.72	0.79	0.74	0.70	0.77
	Polar Type 4	ResNext-101	0.71	0.71	0.67	0.82	0.66	0.58	0.84
MS EBUS data	MS 1-2-3	ShuffleNet-V2	0.69	0.66	0.62	0.71	0.63	0.55	0.76
	MS 1-2-4	ResNet-101	0.69	0.66	0.59	0.75	0.62	0.50	0.82
	MS 2-3-4	DenseNet-201	0.72	0.71	0.70	0.71	0.73	0.71	0.72
PMS EBUS data	PMS 1-2-3	ResNext-101	0.69	0.67	0.62	0.70	0.65	0.57	0.76
	PMS 1-2-4	ResNext-50	0.74	0.64	0.59	0.75	0.61	0.54	0.74
	PMS 2-3-4	MobileNet-V2	0.78	0.74	0.72	0.79	0.70	0.66	0.81
